# Characterization of Biomarker Levels in Crimean–Congo Hemorrhagic Fever and Hantavirus Fever with Renal Syndrome

**DOI:** 10.3390/v11080686

**Published:** 2019-07-26

**Authors:** Miša Korva, Katarina Resman Rus, Miša Pavletič, Ana Saksida, Nataša Knap, Mateja Jelovšek, Katja Strašek Smrdel, Xhevat Jakupi, Isme Humolli, Jusuf Dedushaj, Miroslav Petrovec, Tatjana Avšič-Županc

**Affiliations:** 1 Institute of Microbiology and Immunology, Faculty of Medicine, University of Ljubljana, 1000 Ljubljana, Slovenia; 2 National Institute of Public Health of Kosovo, 10000 Pristina, Serbia

**Keywords:** VHF, CCHF, HFRS, cytokines, biomarkers

## Abstract

Hemorrhagic fever with renal syndrome (HFRS) and Crimean-Congo hemorrhagic fever (CCHF) are important viral hemorrhagic fevers (VHF), especially in the Balkan region. Infections with Dobrava or Puumala orthohantavirus and Crimean-Congo hemorrhagic fever orthonairovirus can vary from a mild, nonspecific febrile illness, to a severe disease with a fatal outcome. The pathogenesis of both diseases is poorly understood, but it has been suggested that a host’s immune mechanism might influence the pathogenesis of the diseases and survival. The aim of our study is to characterize cytokine response in patients with VHF in association with the disease progression and viral load. Forty soluble mediators of the immune response, coagulation, and endothelial dysfunction were measured in acute serum samples in 100 HFRS patients and 70 CCHF patients. HFRS and CCHF patients had significantly increased levels of IL-6, IL-12p70, IP-10, INF-γ, TNF-α, GM-CSF, MCP-3, and MIP-1b in comparison to the control group. Interestingly, HFRS patients had higher concentrations of serum MIP-1α, MIP-1β, which promote activation of macrophages and NK cells. HFRS patients had increased concentrations of IFN-γ and TNF-α, while CCHF patients had significantly higher concentrations of IFN-α and IL-8. In both, CCHF and HFRS patients’ viral load significantly correlated with IP-10. Patients with fatal outcome had significantly elevated concentrations of IL-6, IFN-α2 and MIP-1α, while GRO-α, chemokine related to activation of neutrophils and basophils, was downregulated. Our study provided a comprehensive characterization of biomarkers released in the acute stages of CCHF and HFRS.

## 1. Introduction

Viral hemorrhagic fevers (VHF) are an etiologically diverse group of zoonoses with common pathophysiology. Clinical manifestations of infections vary from asymptomatic or nonspecific febrile illness that can progress to hypovolemic shock and multi-organ failure and death. Two important causative agents of VHF are present in the Balkan area: Crimean-Congo hemorrhagic fever orthonairovirus (CCHFV) and orthohantavirus Dobrava (DOBV) and Puumala (PUUV).

Crimean-Congo hemorrhagic fever (CCHF) is a potentially fatal disease with reported a fatality rate of up to 30%. Cases of CCHF are reported in Kosovo, Eastern Europe, Africa, Asia, and the Middle East [1,2,3,4]. The causative agent, CCHFV, is a negative-stranded RNA virus that belongs to the *Nairoviridae* family of the order *Bunyavirales*. The virus is transmitted through bites of infected *Ixodid* ticks, mainly *Hyalomma* spp., or via direct contact with blood or tissues of viraemic hosts [1,2,5]. Infection in humans is characterized by a febrile illness with headache, myalgia, and petechial rash, frequently followed by a hemorrhagic state with necrotic hepatitis. The acute stage of the disease in survivors usually lasts from 15 to 20 days and is followed by a convalescent period, characterized by prolonged weakness and confusion [1,2].

Pathogenic orthohantaviruses are geographically widespread zoonotic agents from the *Hantaviridae* family of the order *Bunyavirales*. They are genetically and evolutionally closely linked to their small mammal natural hosts [6]. The main transmission route of infection from rodents to humans occurs via inhalation of virus-contaminated aerosols from rodent excreta (urine, saliva or feces). Infection in humans can manifest in two primary forms, Hemorrhagic fever with renal syndrome (HFRS), endemic in Europe and Asia, or Hantavirus pulmonary syndrome (HPS), endemic in the Americas. The clinical spectrum of human infections ranges from asymptomatic infection to severe disease with fatal outcome, partly dependent on the causative virus and partly on host genetic and immune factors [6,7]. The onset of the disease symptoms is abrupt, with fever accompanied by myalgia, headache, transient myopia, nausea, vomiting, diarrhea, abdominal pain, back pain, flushed face, and dizziness [8,9,10]. Although acute kidney injury is a distinctive feature of HFRS, various different clinical manifestations can develop, with pulmonary, hemorrhagic, pancreatobiliary, central nervous system, endocrine, and cardiovascular events [6,11].

Despite many research attempts, the pathogenesis of VHF remains largely unknown. There are several reasons for that: in vitro and in vivo research requires high containment facilities. First, there are no easily accessible animal models, and there is a limited number of patients, most often hospitalized in a remote area. Two VHF, present in the Balkan region, share common pathophysiological and clinical manifestations such as coagulopathy, thrombocytopenia, vascular permeability and hemorrhages. It is thought that the course and outcome of the disease depends on the viral load, host genetic factors and host immune response [12,13,14,15,16]. Despite the knowledge gap in the pathogenesis of VHF, the cytokine storm (i.e., uncontrolled release of cytokines and chemokines) is largely accepted [17,18].

In the present study, we enrolled 170 patients with CCHF or HFRS and measured the levels of cytokines and chemokines associated with innate, adaptive Th1, Th2 responses, regulatory T immune response and those involved in endothelial dysfunction and coagulopathy as the major clinical signs of the VHF. The aim of the study was to characterize biomarkers involved in the pathogenesis of both VHF.

## 2. Materials and Methods

### 2.1. Ethics Statement

The study was performed retrospectively and no additional sample was taken for the purpose of the study. The use of HFRS samples was approved by the Republic of Slovenia National Medical Ethics Committee (no. 69/03/12; 30/04/15). Collection of CCHF serum samples was part of the CCH Fever network (Collaborative Project) supported by the European Commission under the Health Cooperation Work Programme of the 7th Framework Programme (grant agreement no. 260427). Research was approved by the National Medical Ethics Committee of the Republic of Kosovo (no. 05-3193/2). The study was conducted according to the Declaration of Helsinki, we followed the Oviedo Convention on Human Rights and Biomedicine and Slovenian Code of Medical Ethics.

### 2.2. Patients

A total of 100 HFRS (23 female and 77 male) and 70 CCHF patients (24 female and 45 male) were included in the study. All HFRS patients were hospitalized in Slovenian hospitals between the years 2000 and 2014. The diagnosis of HFRS was based on clinical findings (at least two out of three: fever >38 °C, acute kidney injury, thrombocytopenia) and was confirmed serologically and molecularly as described in detail previously [8,19]. Patients’ blood samples were obtained at the time of hospitalization (median 2 days; Table 1), when the clinical diagnosis was confirmed with laboratory tests. According to the collected anamnestic data none of the patients received any treatment before blood collection. The serum was separated from blood cells and serum aliquots were stored at −80 °C until further use. Among HFRS patients, 50 were infected with DOBV (genotype Dobrava) and 50 infected with PUUV. Based on the disease severity and outcome, patients were distributed into three groups: patients with fatal outcome (*n* = 3), patients with severe disease (*n* = 51) and patients with mild disease (*n* = 49).

All 70 CCHF patients were treated at the Infectious Disease Clinic, University Clinical Center of Kosova, Pristina, Kosovo between the years 2001 and 2011. Clinical diagnosis of CCHF was confirmed with serological and molecular tests as described previously in detail [13,14]. Patients’ blood samples were obtained at the time of hospitalization for laboratory diagnostics (median two days; Table 1). According to the collected anamnestic data none of the patients received any treatment before blood collection. Serum was separated from blood cells and aliquots were stored at −80 °C until further use. Detailed medical charts were obtained for 57 CCHF patients. Based on disease progression and the outcome patients were distributed into three groups: patients with fatal outcome (*n* = 14), patients with severe disease (*n* = 18) and patients with moderate disease (*n* = 25).

Additionally, 30 healthy age- and gender-matched controls were also enrolled in our study. Their blood samples were processed and stored as described for patients’ samples. The study was done retrospectively. All enrolled subject have signed inform consent for the studies.

### 2.3. Cytokines and Chemokines

Concentrations of 40 cytokines/chemokines were measured in acute serum samples (first seven days after onset of symptoms) with seven different Human Cytokine/Chemokine Panels (HCYTOMAG-60K, HCYP3MAG-63K, HCVD2MAG-67K, HCVD3MAG-67K, HCVD4MAG-67K, HSP1MAG-63K and HAGP1MAG-12K; Milliplex, Merck Millipore, Burlington, MN, USA) on a MagPix instrument (Luminex, Austin, TX, USA). To minimize inter-assay variation, all measurements in a single panel were performed on the same day in one complete experiment according to the manufacturer’s instructions. All samples were previously aliquoted and diluted to a final concentration 1:5. For all plates in a single panel, simultaneous analysis was done with Milliplex Analyst 5.1 software. In the study, we have investigated cytokines/chemokines associated with innate (granulocyte-colony stimulating factor (G-CSF), granulocyte-macrophage colony-stimulating factor (GM-CSF), growth-regulated oncogene-alpha (GRO-α/CXCL-1), interferon alpha 2 (IFN-α2), interleukin 1-alpha (IL-1α), IL-1β, interleukin-1 receptor antagonist (IL-1RA), IL-6, IL-8, IL-29, monocyte chemoattractant protein 1 (MCP-1/CCL2), MCP-3/CCL7, macrophage colony-stimulating factor (M-CSF), macrophage inflammatory protein 1-alpha (MIP-1α), MIP-1β/CCL4, tumor necrosis factor alpha (TNF-α)), adaptive Th1 (IFN-γ, IL-12p70, IL-12p40, IP-10), adaptive Th2 (IL-4, IL-5), regulatory T immune response (IL-10) and those involved in endothelial dysfunction and coagulopathy (Angiopoietin-2, Fibrinogen, d-dimer, plasminogen activator inhibitor (PAI-1), platelet factor 4 (PF4), soluble CD40 ligand (sCD40L), sE-Selectin, sL-Selectin sP-Selectin, soluble intracellular adhesion molecule sICAM-1, soluble vascular adhesion molecule sVCAM-1, soluble platelet endothelial cell adhesion molecule-1 (sPECAM-1), Thrombomodulin (TM), Tissue factor (TF), VEGF A, von Willebrand factor (vWF), von Willebrand factor-cleaving protease (ADAMTS13)).

### 2.4. Statistical Analyses

Statistical calculations and analysis were performed in GraphPad Prism 8 (GraphPad Software, La Jolla, CA). Statistical analysis values above and below the upper and lower end of the standard cure, were considered as maximum and minimum values, respectively. Values above the maximum were measured only in CCHF fatal cases in two cytokines: M-CSF (*n* = 9) and Angiopoietin-2 (*n* = 5). Biomarkers with >50% of measurements out of range were excluded from the analysis. To analyze the normal distribution of data the D’Agostino-Pearson normality test was performed. The identification of outliers was performed using Dixon’s Q test. Statistically significant differences in the serum concentrations of cytokines between severe and mild DOBV or PUUV infection were determined using the Mann–Whitney test (P). The Kruskal–Wallis test was used to determine differences among groups in comparison between moderate, severe and fatal cases of CCHF and HFRS and for comparison of cytokine levels among DOBV and PUUV infected patients and control groups. Correlations between biomarkers and viral loads of HFRS patients and CCHF patients were done with a nonparametric two-tailed Spearman correlation test with a 95% confidence interval. To control for the false discovery rate, P values were adjusted for multiple comparisons. Differences with *P* < 0.05 were considered significant.

## 3. Results

Study population included 170 VHF patients infected with orthohantaviruses or CCHFV. Among 100 HFRS patients, 50 were infected with DOBV (genotype Dobrava) and 50 infected with PUUV. Serum samples were collected during the acute phase of infection, median on the 2nd day of hospitalization, which is between 3–11 days after self-reported onset of symptoms (Table 1). Medical charts were collected for each patient and based on selected clinical and laboratory criteria [19,20] they were categorized into mild or severe groups. Three patients had fatal outcome of the disease, and they were all infected with DOBV. The criteria for severe HFRS were: 1. the need for dialysis, or 2. the lowest systolic blood pressure <90 mm Hg and/or clinical signs of shock, or 3. thrombocytopenia <50 × 10^9^/L and 4. the presence of a) bleeding and/or b) renal failure manifested with oliguria (diuresis <500 mL/day) and/or >4× higher than the upper normal level of urea or creatinine. Out of 100 HFRS patients, 48 patients (23 infected with DOBV, 25 infected with PUUV) fulfilled the criteria for severe disease and 49 patients were found to have mild disease (24 infected with DOBV, 25 infected with PUUV). In all HFRS patients, viral RNA was detected in the acute sample. The viral load was ranging from 0.2–7.6 log RNA copies/mL for PUUV infected patient and from 1.3–8.3 log RNA copies/mL for DOBV infected patients. On the contrary, IgM and IgG antibodies were detected in 98% and 46% of HFRS patients, respectively (Table 1).

Among 70 patients with acute CCHF infection, 14 patients had fatal outcome of the disease. We were able to collect detailed medical charts for 43/56 surviving CCHF patients and based on selected clinical and laboratory criteria patients were categorized into moderate or severe groups. Surviving patients fulfilling at least three of the following criteria were defined as having severe CCHF: The presence of profound hemorrhagic manifestations (blood transfusion required), increased serum creatinine values, increased serum transaminase values, and hypotension (blood pressure <100/60 mm Hg) [14]. None of the patients included in the study received ribavirin treatment. Out of 43 CCHF patients, 25 patients had moderate and 18 patients had severe disease. Also, all CCHF patients enrolled in our study were found to be PCR positive, with viral load ranging from 2.0–10.0 log RNA copies/mL (Table 1). As expected, the highest viral loads were detected in CCHF patients with fatal outcome. Antibody response was measured only in the minority of CCHF patients, IgM antibodies were found in 33% patients and IgG in only 7.1% of patients (Table 1). Serum samples were collected during the acute phase of infection, median on the 2nd day of hospitalization, which is between 2–10 days after self-reported onset of symptoms (Table 1).

Bleeding and profound endothelial dysfunction with capillary leakages, the hallmark of VHF pathology, have been reported in 10% of HFRS patients included in the study (six infected with DOBV and four infected with PUUV) and in 45% of CCHF patients.

### 3.1. Comparison of Cytokine and Chemokine Levels in HFRS Patients

**Figure 1:**
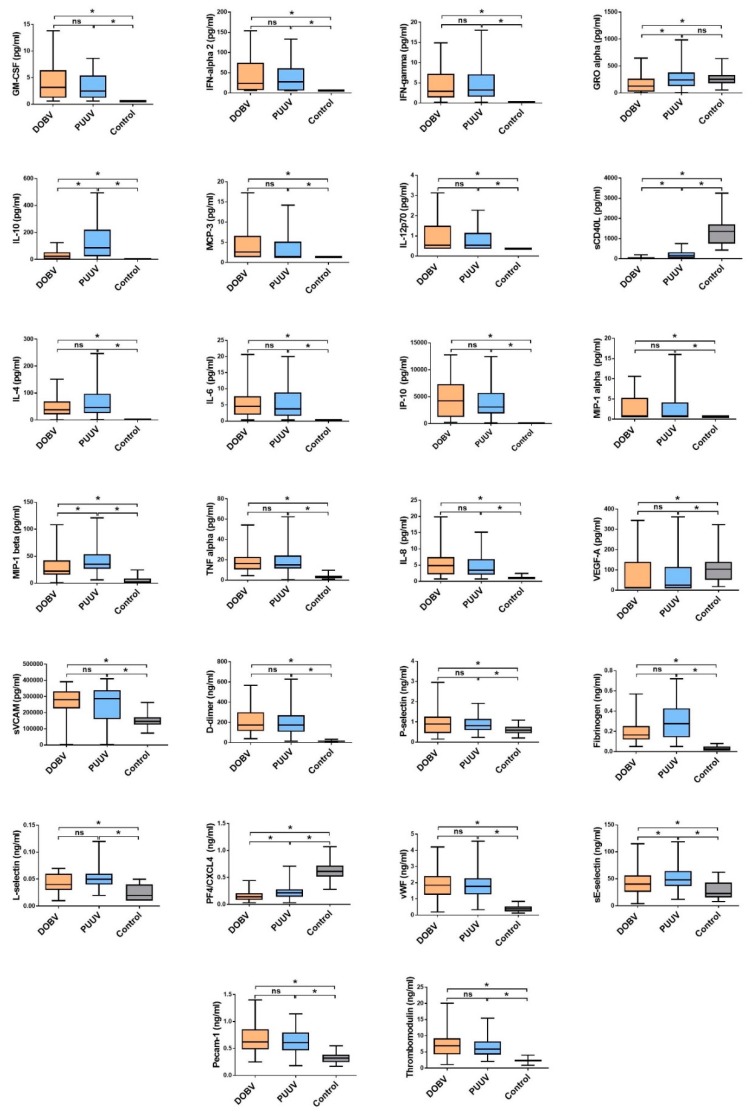
The cytokine and chemokine concentrations with significant differences in HFRS patients (DOBV, *n* = 50; PUUV, *n* = 50) in comparison to control group (*n* = 30). The samples were obtained median 6 days after the onset of symptoms. The statistically significant differences are marked with * (*p* < 0.05). ns= not statistically significant. The boxes represents medians with interquartile ranges; the whiskers depict minimum and maximum values (range). Identified outliers are not included in the figures above: IFN-γ (DOBV *n* = 4; PUUV *n* = 3), MCP-3 (DOBV *n* = 6; PUUV *n* = 5), IL-4 (DOBV *n* = 5; PUUV *n* = 5), MIP-1α (DOBV *n* = 3; PUUV *n* = 8), IL-8 (DOBV *n* = 7; PUUV *n* = 4), VEGF-A (PUUV *n* = 6) and Fibrinogen (DOBV *n* = 8; PUUV *n* = 2).

In acute serum samples of HFRS patients 26/40 cytokines and chemokines differ significantly in comparison to healthy controls. Six cytokines (IL-1α, IL-1β, IL-5, IL-12p40, IL-29 and M-CSF) were below the lower limit of detection in >60% of patients and thus were not evaluated further. Significantly increased cytokines and chemokines in HFRS patients were those predominantly associated with innate and adaptive Th1 immune response (IFN-γ, IL-1RA, IL-6, IL-8, IL-12p70, IP-10 and TNF-α) in comparison to the control group (Figure 1). In addition, HFRS patients had higher concentrations of serum MCP-1, MCP-3, MIP-1α and MIP-1β, which regulate migration of monocytes and activation of macrophages, and NK cells at the site of infection. Also, GM-CSF, an immune modulatory cytokine that has the ability to induce proliferation of granulocyte and macrophage populations from precursor cells and to activate T-cell immune response, was significantly higher in HFRS patients in comparison to the control group (Figure 1).

**Figure 2:**
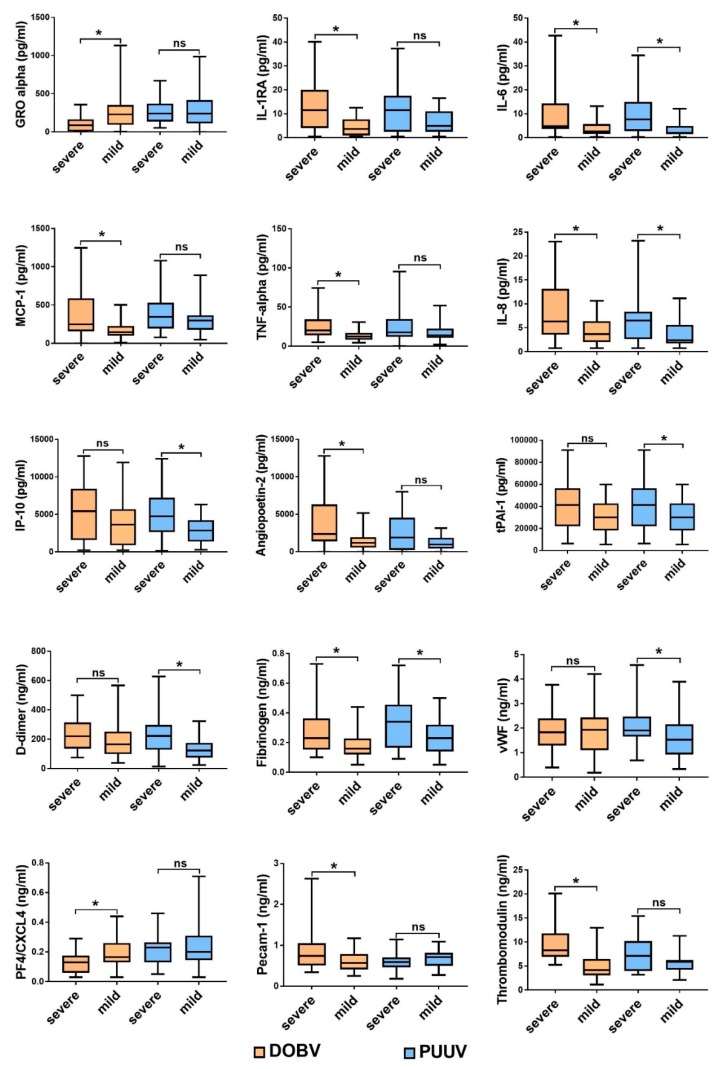
The cytokine and chemokine concentrations with significant differences in patients infected with DOBV (severe *n* = 25; mild *n* = 25) or PUUV (severe *n* = 25; mild *n* = 25), with regard to the disease progression and outcome. The statistically significant differences are marked with * (*p* < 0.05). ns= not statistically significant. The boxes represents medians with interquartile ranges; the whiskers depict minimum and maximum values (range). Identified outliers are not included in the figures above: IL-6 (DOBV severe *n* = 4; DOBV mild *n* = 1; PUUV severe *n* = 1; PUUV mild *n* = 4), MCP-1 (DOBV severe *n* = 1; DOBV mild *n* = 4; PUUV severe *n* = 5; PUUV mild *n* = 4), IL-8 (DOBV severe *n* = 5; DOBV mild *n* = 3), D-dimer (PUUV mild *n* = 3), Fibrinogen (DOBV severe *n* = 5; DOBV mild *n* = 1; PUUV mild *n* = 2).

Patients with PUUV had higher levels of anti-inflammatory IL-10 and GRO-α than those infected with DOBV (Figure 1). On the other hand, DOBV infected patients with severe disease had significantly elevated levels of IL-6, IL-8, IL1-RA, TNF-α and MCP-1, while PUUV infected patients with severe disease also had higher levels of IP-10 in comparison to those with mild disease (Figure 2).

Additionally, we have investigated factors indicating endothelial dysfunction in patients with HFRS. HFRS patients had significantly increased serum concentrations of soluble fractions of adhesion molecules (sE-Selectin, sL-Selectin and sP-Selectin) and sVCAM-1, PECAM-1 which facilitate the entry of leukocytes into inflamed tissues, in comparison to the control group. In addition to that, patients with HFRS, especially severe cases, also had significantly higher concentrations of Angiopoietin-2, vWF, Fibrinogen, TM and TF in comparison to control group, suggesting changes in hemostasis (Figure 2). On the contrary, sCD40 ligand, which is considered to contribute to the promotion of prothrombotic responses and production of angiogenesis-associated factor, was significantly downregulated in HFRS patients in comparison to controls or to mild disease, regardless of the virus in question (Figure 2).

### 3.2. Comparison of Cytokine and Chemokine Levels in CCHF Patients

**Figure 3:**
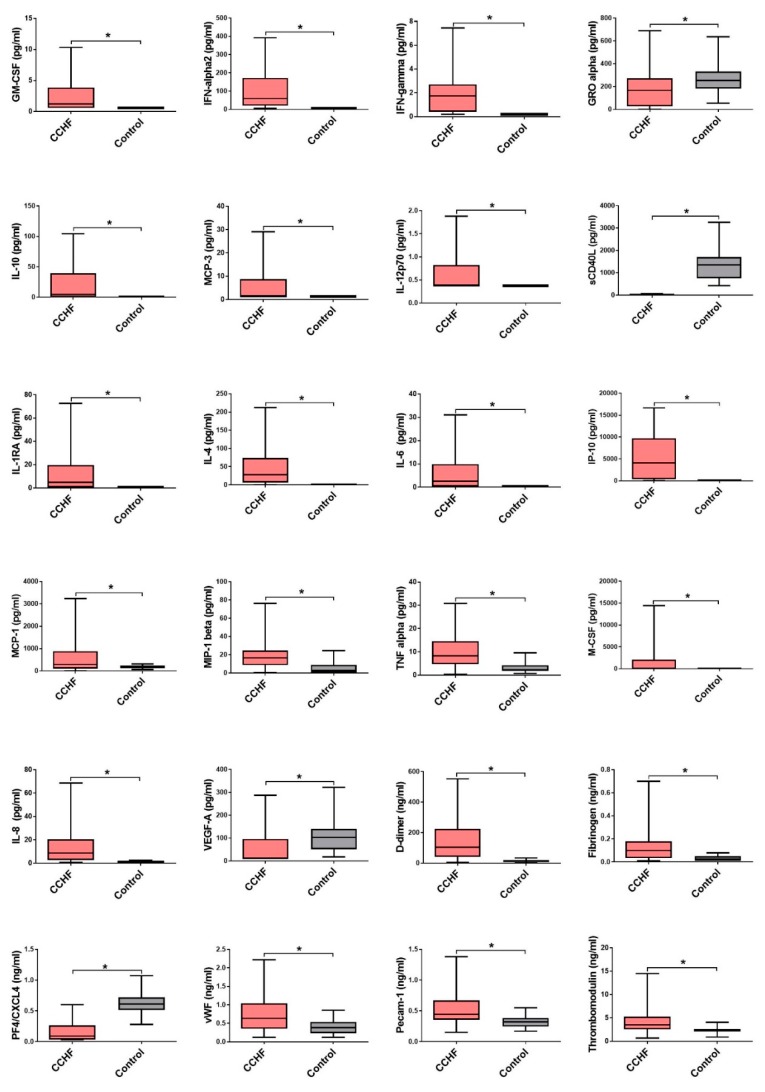
The cytokine and chemokine concentrations with significant differences in CCHF patients (*n* = 70) in comparison to control group (*n* = 30). The samples were obtained median six days after the onset of symptoms. The statistically significant differences are marked with * (*p* < 0.05). ns= not statistically significant. The boxes represents medians with interquartile ranges; the whiskers depict minimum and maximum values (range). Identified outliers are not included in the figures above: GM-CSF (CCHF *n* = 5), IFN-α (CCHF *n* = 5), IFNγ (CCHF *n* = 1), GRO-α (CCHF, *n* = 6), IL-10 (CCHF *n* = 5), MCP-3 (CCHF *n* = 8), IL-4 (CCHF *n* = 8), IL-6 (CCHF *n* = 1), MCP-1 (CCHF *n* = 1), MIP-1β (CCHF *n* = 7), M-CSF (CCHF *n* = 1), VEGF-A (CCHF *n* = 6), TM (CCHF *n* = 5).

**Figure 4:**
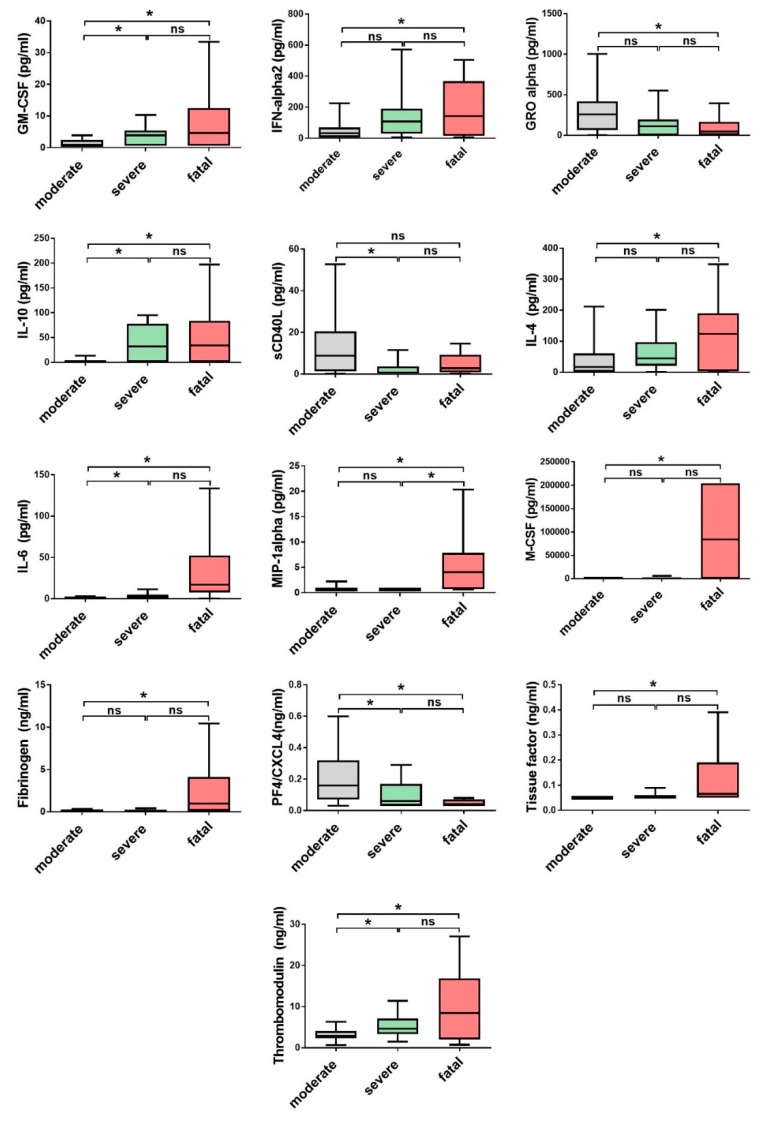
The cytokine and chemokine concentrations with significant differences in CCHF patients, with regard to the disease progression and outcome (moderate *n* = 24; severe *n* = 18; fatal *n* = 14). The statistically significant differences are marked with * (*p* < 0.05). ns= not statistically significant. The boxes represents medians with interquartile ranges; the whiskers depict minimum and maximum values (range). Identified outliers are not included in the figures above: GM-CSF (moderate *n* = 3; severe *n* = 1), IFN-α (moderate *n* = 2; fatal *n* = 1), GRO-α (moderate *n* = 2; severe *n* = 3), IL-10 (moderate *n* = 4; fatal *n* = 1), IL-4 (moderate *n* = 3; severe *n* = 2), IL-6 (moderate *n* = 7; severe *n* = 3; fatal *n* = 1), MIP-1α (moderate *n* = 6; severe *n* = 4; fatal *n* = 2).

In acute serum samples of CCHF patients, 25/40 cytokines and chemokines significantly differ in comparison to healthy control. The levels of five cytokines (IL-1α, IL-1β, IL-5, IL-12p40 and IL-29) were below the lower limit of detection in >60% of patients and thus were not evaluated further. Predominantly CCHF patients had significantly elevated levels of cytokines and chemokines associated with innate and adaptive Th1 immune response (GM-CSF, IFN-α2, IFN-γ, IL-1RA, IL-6, IL-8, MCP-1, MCP-3, M-CSF, MIP-1β, TNF-α, IL-12p70, IP-10) in comparison to the control group (Figure 3). Regardless of the disease course and outcome most of the patients had significantly elevated levels of IL-12p70, IFN-γ and IP-10, an IFN-γ inducible chemokine that attracts monocytes, macrophages, T cells, NK cells and dendritic cells. Among fatal cases, significantly elevated concentrations of IL-6 and IL-10 were detected in comparison to the survivors (Figure 4). Also, fatal cases have higher levels of cytokines related to macrophage and granulocyte proliferation (MIP-1α, GM-CSF, M-CSF), while GRO-α, chemokine related to activation of neutrophils and basophils, was higher in patients with moderate disease progression (Figure 4).

Since most of the patients with CCHF have hemorrhage related disorders (petechia, ecchymosis and melena), markers of endothelial dysfunction and coagulopathy were also investigated. CCHF patients had significantly elevated levels of d-dimer, fibrinogen, vWF, PECAM-1 and TM compared to control group. Fatal CCHF patients had significantly higher levels of fibrinogen and TM, which are involved in disseminated intravascular coagulation (DIC). On the contrary, both markers of platelet activation, soluble effector molecule sCD40L and PF4, were higher in patients with moderate diseases progression (Figure 4).

### 3.3. Soluble Biomarkers Involved in the Pathogenesis of both VHF

**Figure 5:**
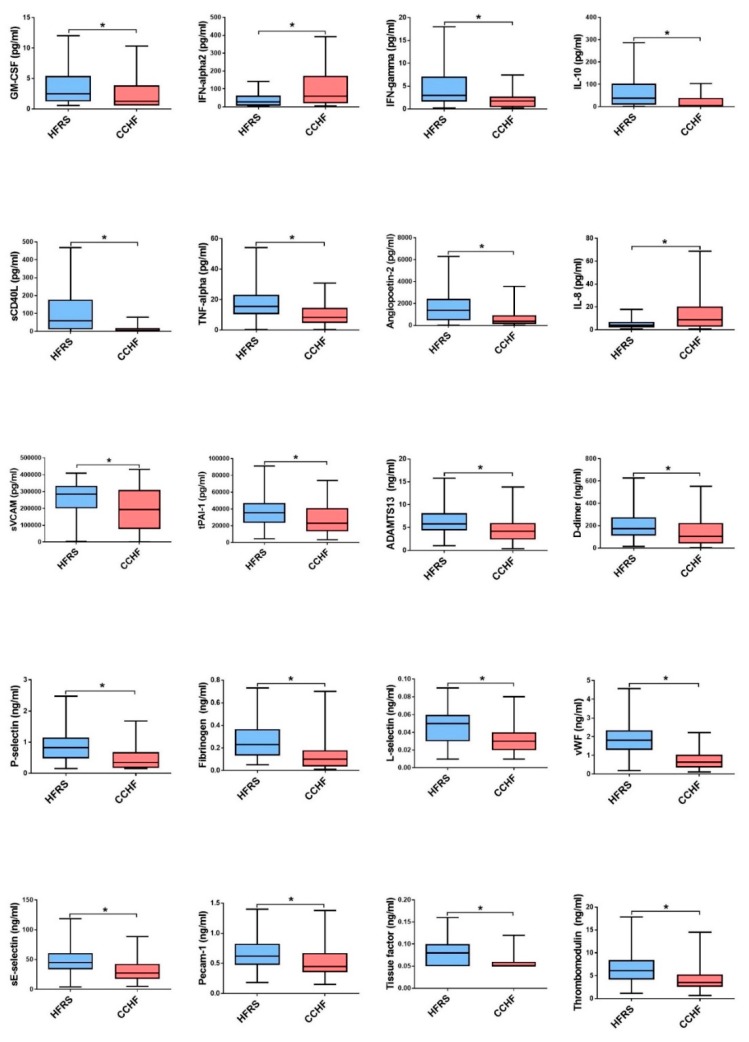
The cytokine and chemokine concentrations with significant differences between in HFRS (*n* = 100) and CCHF (*n* = 70) patients. The statistically significant differences are marked with * (*p* < 0.05). ns= not statistically significant. The boxes represents medians with interquartile ranges; the whiskers depict minimum and maximum values (range). Identified outliers are not included in the figures above: GM-CSF (HFRS *n* = 8; CCHF *n* = 5), IFN-α2 (HFRS *n* = 4; CCHF *n* = 5), IFN-γ (HFRS *n* = 8; CCHF *n* = 1), IL-10 (HFRS *n* = 8; CCHF *n* = 5), TNF-α (HFRS *n* = 6; CCHF *n* = 3), Angiopoetin-2 (HFRS *n* = 9; CCHF *n* = 1), D-dimer (HFRS *n* = 1; CCHF *n* = 4), P-selectin (HFRS *n* = 4; CCHF *n* = 7), pCAM-1 (HFRS *n* = 4; CCHF *n* = 2), TF (HFRS *n* = 6; CCHF *n* = 8), TM (HFRS *n* = 2; CCHF *n* = 5).

As both, CCHFV and orthohantaviruses can cause a wide spectrum of the disease, from asymptomatic disease to a fatal outcome, we wanted to compare cytokines and chemokines levels response in both groups. Interestingly, in our study HFRS patients had a more diverse and higher concentrations of most soluble biomarkers in comparison to the CCHF patients. HFRS patients had higher concentrations of TNF-α and IFN-γ, where CCHF patients had significantly higher concentrations of IFN-α2 and IL-8 (Figure 5). Also, HFRS patients had higher concentrations of serum MIP-1α, MIP-1β, which promote activation of macrophages and NK cells. What is more, patients with HFRS had higher markers of endothelial dysfunction (Angiopoetin-2, sVCAM-1, PAI-1, ADAMTS13, d-dimer, sP-Selectin, fibrinogen, vWF, sE-Selectin, sPECAM-1, TF and TM) than CCHF patients.

**Figure 6:**
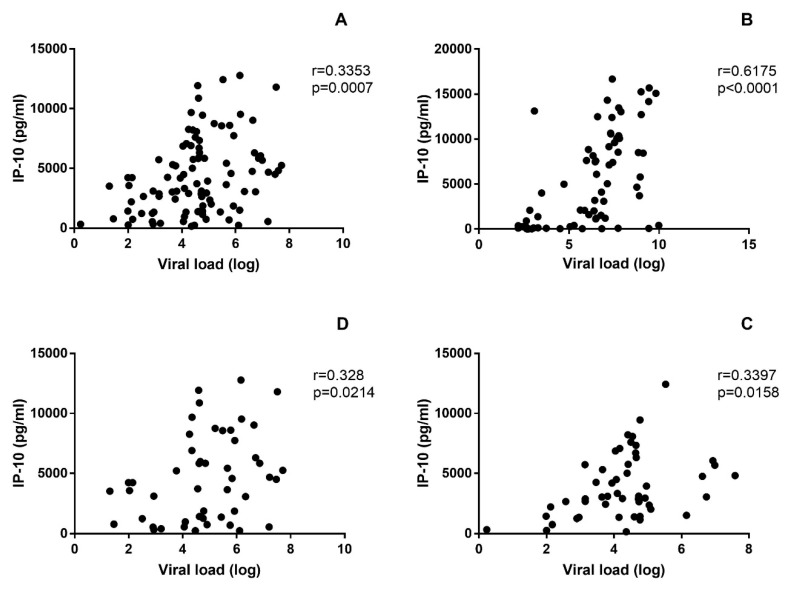
Correlation between biomarker IP-10 and viral loads of HFRS patients (**A**), CCHF patients (**B**), patients infected with DOBV (**C**) and patients infected with PUUV (**D**). Nonparametric two-tailed Spearman correlation test with 95% confidence interval was used.

In addition, we have explored a possible correlation between viral load and different measured biomarkers. The strongest correlation was found between viral load and IP-10 in both CCHF (r = 0.62, *p* < 0.0001) and HFRS patients (r = 0.34, *p* = 0.0007) (Figure 6). Positive correlation between viral load and IFN-α2 (r = 0.25 *p* = 0.0118) was found in HFRS patients, but not in CCHF patients. Moreover, positive correlations between viral load and endothelial dysfunction markers (Angiopoietin-2 (r = 0.45 *p* = 0.0001), PAI-1 (r = 0.43 *p* = 0.0002), ADAMTS3 (r = 0.27 *p* = 0.0266), d-dimer (r = 0.31 *p* = 0.01), sPECAM-1 (r = 0.41 *p* = 0.0007), TF (r = 0.34 *p* = 0.0047) and TM (r = 0.43 *p* = 0.0002)) were found in CCHF patients. In HFRS patients, viral load was only associated with Angiopoietin-2 (r = 0.21 *p* = 0.0394).

## 4. Discussion

In VHF a severe clinical manifestation with a fatal outcome is proposed to be a result of uncontrolled release of cytokines and chemokines (i.e., a cytokine storm) [17,18]. The innate immune response represents the first line of defense against various viral infections, including CCHF and HFRS. Secretion of type I cytokines, mainly IFN-α and IFN-β, lead to upregulation of interferon stimulated genes (ISGs) such as MxA, which was shown to inhibit viral replication of CCHF and orthohantaviruses via interaction with the viral nucleocapsid protein [21,22,23,24,25]. It was reported that early activation of IFN I response can be delayed by antagonistic action of CCHFV and orthohantaviruses, thus enabling early replication of viruses and their systemic spread [26,27].

In our study, both CCHF and HFRS, patients had significantly increased cytokines and chemokines associated with strong innate and adaptive Th1 immune response in comparison to the control group. HFRS patients had higher concentrations of TNF-α and IFN-γ, where CCHF patients had significantly higher concentrations of IFN-α2 and IL-8, proposing activation of different immune cells. Studies have shown that TNF-α contributes to hematophagocytosis trough macrophage activation, has an antifibrinolytic action and may disrupt endothelial permeability and integrity [28,29]. In addition, IL-8 is known as the major chemotactic factor for neutrophil granulocytes and was shown to positively correlate with kidney dysfunction in PUUV infected HFRS patients and with fatal cases of CCHF [30,31]. Elevated levels of different pro-inflammatory cytokines, especially in severe and fatal CCHF patients and in severe HFRS patients have already been reported previously [12,14,15,18,19,30,32,33,34,35,36,37,38]. Similar cytokine patterns observed in our study in CCHF and HFRS patients, were seen also in patients with Ebola virus disease (EVD) where elevated levels of IL-1β, IL-1RA, IL-6, IL-8, IL-15, MIP-1α, MIP-1β, MCP-1, MCP-3, IP-10 and eotaxin were associated with fatal outcome [39,40].

Interleukine-6 and TNFα were shown to cause endothelial barrier dysfunction via the protein kinase C pathway resulting in ultrastructural changes in distribution of tight junctions, intracellular actin disorganization and cellular morphological changes [41,42]. We have measured significantly increased concentrations of IL-6 in fatal CCHF patients in comparison to the survivors. Increased serum levels of IL-6 were reported in other cases of VHF, such as EVD and Lassa fever [43,44,45]. Besides, our results support the previous findings [18,30], that higher levels of cytokines MIP-1α, GM-CSF, M-CSF, which are all related to macrophage and granulocyte activation, are significantly increased in fatal CCHF patients. However, higher levels of MIP-1β, chemoattractant and activator of NK cells, have been reported in CCHF patients with milder disease [30], while in our study there was no correlation with disease progression.

A recent study showed, that high concentrations of IP-10 and MCP-1 strongly correlate with high viral load and severity of the disease in CCHF patients [18,46,47]. Most CCHF patients included in our study had significantly elevated levels of IP-10 regardless of the disease course or outcome. In HFRS patients, significantly higher levels of IP-10 were observed in PUUV infected patients with severe disease in comparison to those with mild disease. Similar results were seen in DOBV infected patients, but the difference between severe and mild disease was not significant. Elevated levels of IP-10 were also previously reported in Hantaan infected patients with severe disease [48]. Above that, we have discovered a strong positive correlation between viral load and IP-10 in both CCHF and HFRS patients. Positive correlation between viral load and IFN-α2 was established in HFRS patients, but not in CCHF patients. We have shown that DOBV infected patients with severe disease had significantly higher levels of MCP-1, MCP-3 and MIP-1α, which regulate migration of monocytes, macrophages and NK cells at the site of infection. Also, MCP-1, induced by VEGF, is known to cause vascular leakage in vivo due to reduced tight junctions between vascular endothelium cells [49,50]. Increased levels of MCP-1 were observed in Dengue hemorrhagic fever patients with increased vascular permeability [50].

It is thought that elevated levels of anti-inflammatory IL-10 in early stages of infection impairs Th1 cell response and enables virus replication and spread. It was shown that elevated concentration of IL-10 correlate with viral load and severity of the disease in HFRS patients [19,35]. Similar findings have also been conveyed for fatal CCHF patients [14], but not for survived severe cases [2,15]. Elevated levels of IL-10 were reported in Dengue hemorrhagic fever and in fatal cases of EVD too [43,51]. In our study, HFRS and CCHF patients had higher levels of IL-10 in comparison to the control group. When comparing both VHF, surprisingly higher IL-10 concentrations were measured in HFRS patients in comparison to CCHF patients. Another anti-inflammatory cytokine, IL-4, an important activation marker of Th2 immune response, was shown to be upregulated in early phase of HFRS [46]. Some studies reported that IL-4 was significantly upregulated also in the late phase of severe HFRS, while others claimed different results [16,36]. IL-4, was shown to be also increased in patients with Dengue hemorrhagic fever [52,53]. In our study, significantly higher concentrations of IL-4 were measured in HFRS infected patients in comparison to the control group, but there was no difference between the causative agents or disease severity. Concentrations of IL-4 were also significantly higher in CCHF patients, where highest levels were measured in patients with a fatal outcome.

In both investigated VHF, endothelial dysfunction with temporary capillary leakage, which can result in tissue edema and organ failure is the major hallmark of the disease. In HFRS, the pathogenesis is induced without any obvious cytopathology in the capillary endothelium. Thus, it implies that vascular leakage is more influenced by host immune response than virus characteristics. This is conferred with our results where, in general, patients with HFRS had higher levels of markers indicating endothelial dysfunction than CCHF patients, despite the fact that CCHF has a fatality rate of up to 30% and that most severe or fatal patients present with hemorrhages. In our study, HFRS patients had significantly increased serum concentrations of sE-Selectin, sL-Selectin and sP-Selectin and sVCAM-1, PECAM-1, which facilitate the entry of leukocytes into inflamed tissues. Increased expression of ICAM-1 and sVCAM-1 molecules by endothelial cells of blood vessels affects rolling, adhesion and transmigration of leucocytes and can result in local inflammatory response that can lead to endothelial cell damage and bleeding [54,55]. In addition to that, patients with HFRS, especially severe cases, also had significantly higher concentrations of Angiopoietin-2, vWF, Fibrinogen, TM and TF in comparison to control group, suggesting changes in hemostasis. Angiopoietin-2 is a proangiogenic factor that has recently been implicated in the direct control of inflammation-related signaling pathways [56]. In our study, a positive correlation between viral load and Angiopoietin-2 was observed for HFRS and CCHF patients. On the contrary, Angiopoetin-1 was reported to inhibit hantavirus directed endothelial cell permeability [57]. Besides, Angiopoietin-2 several other endothelial dysfunction markers, like PAI-1, ADAMTS3, d-dimer, sPECAM-1, TF and TM were found to correlate with viral load, but only in CCHF patients. CCHF patients had significantly elevated levels of d-dimer, fibrinogen, vWF, PECAM-1 and TM compared to control group. Fatal CCHF patients had significantly higher levels of fibrinogen and TM, which are involved in DIC. Endothelial dysfunction and disseminated intravascular coagulation, accompanied with increased expression of sICAM-1, sVCAM-1 and TM has also been reported in fatal EVD cases [40,58].

On the other hand, higher concentrations of two inflammatory factors, GRO-α and sCD40L, seem to play a protective role in vascular damage and hemorrhaging in VHF. Increased levels of GRO-α were reported in milder cases of Dengue infections without blood leakage [59,60]. In our study, patients with PUUV had higher levels of GRO-α than those infected with DOBV, thus they were able to more efficiently regulate the immune response resulting in milder disease. Even more, fatal CCHF patients and severe DOBV patients had significantly lower concentrations of GRO-α in comparison to patients with milder disease. Similar findings were reported concerning sCD40L, an important proinflammatory factor released by activated platelets. HFRS and CCHF patients had significantly lower levels of sCD40 ligand in comparison to controls. The lowest concentrations of sCD40L were measured in CCHF patients with fatal outcome, who also had the most pronounced thrombocytopenia. Significantly, increased levels of sCD40L were reported in milder cases of Dengue hemorrhagic fever and EVD, suggesting a potential protective role of sCD40L in pathogenesis of VHF. However, low sCD40 levels also correlated with low platelet counts that could result in observed decreased concentration of sCD40L in these patients [40,59].

VHF are a group of clinically similar diseases, but the intensity of clinical symptoms can vary from asymptomatic to lethal. The differences in disease progression can only partly be explained by the direct influence of the virus and are severely influenced by the host’s immune response. The majority of the studies in this field have shown that both, low and massive immune response can lead to lethal disease, and only if controlled release of cytokines is produced the viral infection results in a milder disease [61]. The key question now is how to control and modify the release of cytokines in order to limit the impact of the disease; however, the answer to this question is yet to be discovered.

## Figures and Tables

**Table 1:** Antibody response, viral RNA load, self-reported onset of symptoms and day of hospitalization, by disease course and causative agent.

Virus	Disease Course	No. of Enrolled Patients	Self-Reported Onset of Symptoms (Median; Min/Max)	Day of Hospitalization (Median; Min/Max)	Viral load (Median *; Min/Max)	IgG Pos/Neg (Min; Max Titer)	IgM Pos/Neg (Min; Max Titer)
CCHF	moderate	25	7 (2–10)	2 (1–7)	5.6 (2.2–7.9)	2/25(800; 1600)	7/25 (800; >6400)
	severe	18	5 (2–8)	2 (1–4)	6.9 (2.7–8.9)	1/18 (100)	6/18 (1600; 6400)
	fatal	14	4 (2–7)	2 (1–2)	8.9 (2.7–10.0)	0/14	9/14 (400; 3200)
	**∑**	**70**	**6 (2–10)**	**2 (1–7)**	**6.6 (2.0–10.0)**	**5/70 (100; 1600)**	**23/70 (400; >6400)**
PUU	mild	25	6 (3–10)	2 (1–6)	3.7 (0.2–7.0)	15/25 (400; >6400)	25/25 (100; >6400)
	severe	25	6 (3–9)	2 (1–8)	4.6 (2.6–7.6)	13/25 (100; >6400)	24/25 (400; >6400)
	**∑**	**50**	**6 (3–10)**	**2 (1–8)**	**4.4 (0.2–7.6)**	**27/50 (100; >6400)**	**49/50 (100; >6400)**
DOB	mild	25	7 (3–11)	2 (1–8)	4.6 (1.3–8.3)	9/25 (800; 3200)	24/25 (800; >6400)
	severe	22	7 (3–11)	2 (1–6)	5.7 (2.1–7.7)	9/22 (100; >6400)	22/22 (1600; >6400)
	fatal	3	4 (3–4)	2 (1–4)	5.2 (4.3–6.8)	0/3	3/3 (1600; 6400)
	**∑**	**50**	**7 (3–11)**	**2 (1–8)**	**4.8 (1.3–8.3)**	**18/50 (100; >6400)**	**49/50 (800; >6400)**

* determined with quantitative real-time RT-PCR (log RNA copies/mL); text in bold represents the values for the CCHF, PUU, DOB groups regardless of the disease progress.
